# Diagnosis of Biofilm-Associated Peri-Implant Disease Using a Fluorescence-Based Approach

**DOI:** 10.3390/dj9030024

**Published:** 2021-02-27

**Authors:** Geelsu Hwang, Markus B. Blatz, Mark S. Wolff, Liviu Steier

**Affiliations:** 1 Department of Preventive and Restorative Sciences, School of Dental Medicine, University of Pennsylvania, Philadelphia, PA 19104, USA; geelsuh@upenn.edu (G.H.); mblatz@upenn.edu (M.B.B.); mswolff@upenn.edu (M.S.W.); 2 Center for Innovation and Precision Dentistry, School of Dental Medicine, School of Engineering and Applied Sciences, University of Pennsylvania, Philadelphia, PA 19104, USA

**Keywords:** peri-implant diseases, biofilm, fluorescence enhanced theragnosis

## Abstract

Dental implants have become a routine component of daily dental practice and the demand for dental implants is expected to increase significantly in the future. Despite the high success rates of dental implants, failures do occur, resulting in discomfort, rampant destruction of the oral health, or painful and costly surgical replacement of a failed implant. Peri-implant diseases are inflammatory conditions affecting the soft/hard tissues surrounding a functional dental implant. Plenty of experimental evidence indicates that the accumulation of dental plaque at the soft tissue–implant interface and the subsequent local inflammatory response seems to be key in the pathogenesis of the peri-implant mucositis. Such peri-implant–soft tissue interface is less effective than natural teeth in resisting bacterial invasion, enhancing vulnerability to subsequent peri-implant disease. Furthermore, in certain individuals, it will progress to peri-implantitis, resulting in alveolar bone loss and implant failure. Although early diagnosis and accurate identification of risk factors are extremely important to effectively prevent peri-implant diseases, current systematic reviews revealed that a uniform classification and diagnostic methodology for peri-implantitis are lacking. Recent progress on fluorescence-based technology enabled rapid diagnosis of the disease and effective removal of plaques. Here, we briefly review biofilm-associated peri-implant diseases and propose a fluorescence-based approach for more accurate and objective diagnoses. A fluorescence-based diagnosis tool through headlights combined with special-filtered dental loupes may serve as a hands-free solution for both precise diagnosis and effective removal of plaque-biofilms.

## 1. Introduction

Osseointegrated dental implants have become a reliable treatment option for replacing missing teeth and are now a routine component of daily dental practice [1,2]. There has been a large increase in the prevalence of patients receiving dental implants ranging from 0.7% in 2000 to 5.7% in 2016 [3]. Every year, approximately five million implants are placed in the US as per the American Dental Association, and the global dental implant market is expected to reach around $4.5 billion a year by 2022 [3].

Despite the high success rates of dental implants, it is challenging to maintain them in the long term. Many studies reported relatively high frequencies of peri-implant diseases with some variances. For example, one study surveyed 1497 participants with 6283 implants and reported that peri-implant mucositis was found in 63.4% of participants and 30.7% of implants, and peri-implantitis in 18.8% of participants and 9.6% of implants [4]. A survey of periodontists in the US revealed a prevalence of peri-implant mucositis and peri-implantitis in their practices of up to 25% [5]. Another cross-sectional study reported the frequencies of peri-implant mucositis and peri-implantitis, in individuals, as 54% and 28%, respectively [6]. Finally, a meta-analysis of 47 studies summarized that the prevalences of peri-implantitis of weighted mean implant and participant were 9.25% and 19.83%, and those of peri-implant mucositis were 29.48% and 46.83%, respectively [7]. In summary, these studies demonstrate a high incidence of peri-implant diseases among patients three to 18 years after implantation. Inconsistent definitions and vague criteria for diagnosis of peri-implant disease may limit objective studying of the prevalence and extent of peri-implant diseases. A clear definition of peri-implant mucositis and implantitis and the development of improved diagnostic tools is warranted if study outcomes are to be compared. This article briefly reviews biofilm-associated peri-implant diseases and proposes a fluorescence-based approach for more accurate and objective diagnoses.

## 2. Definition of Peri-Implant Diseases

Peri-implant diseases are inflammatory conditions affecting the soft and hard tissues surrounding a dental implant [8,9]. Under healthy conditions, peri-implant soft tissues around implant-supported restorations protect the implant–bone interface around osseointegrated implants against bacterial invasion. However, the soft tissue adjacent to these restorations has been shown to be less effective than that of natural teeth in resisting bacterial invasion due to the lack of a true connective tissue attachment and reduced vascular supply, resulting in enhanced vulnerability to subsequent peri-implant disease [10,11]. Such peri-implant diseases are classified into two categories: peri-implant mucositis and peri-implantitis. While clinical signs of soft tissue inflammation are observed in both forms of peri-implant disease, alveolar bone loss that can potentially lead to implant loss is unique to peri-implantitis [9,12]. Despite peri-implant mucositis being a relatively benign and reversible condition [8,13], it is critical to recognize that, in some individuals, it will progress to peri-implantitis, which may be highly destructive and irreversible. Particularly, smokers or patients with a history of chronic periodontitis or diabetes were shown to be more susceptible to peri-implant diseases [14,15,16,17,18,19,20,21].

## 3. Pathogenesis of Peri-Implant Diseases

Soft mucosal and hard dental tissues are continuously colonized by oral microbial flora. More than 700 species are accounted for oral microbiota, while distinct subsets are found from individual habitats [22,23]. These oral microbial cells form structured communities called biofilms on soft and hard tissues in the oral cavity, exhibiting a highly functionalized microbial organization [24]. These microbial communities are associated with health or disease at distinct oral sites [25]. Changes in the local microenvironment may trigger the overgrowth of pathogenic bacterial species, and in turn, result in a shift in the composition of the biofilm microflora. Ultimately, this may lead to dysbiosis between the resident oral microbiota and the host [26,27], frequently expressing virulent properties.

Dental caries and periodontal diseases are representative oral diseases, both caused by biofilms growing on natural tooth and tooth restoration surfaces. Biofilms on the tooth surface may demineralize the enamel, and dentine, when exposed to dietary carbohydrates such as sugars, results in fermentation and the generation of acids. Periodontal diseases are also attributed to changes in the polymicrobial biofilms accumulated on the tooth surface in the subgingival area. The thickened biofilm results in the destruction of the tooth-supporting (periodontal) tissues as a result of the excessive host-modulated inflammatory response to the biofilm from the juxtaposed gingival tissue. Persistence and progression of a heightened inflammatory response can result in enzymatic destruction of the deeper periodontal tissues that link the tooth surface to the supporting alveolar bone. If untreated, periodontitis will eventually result in tooth loss. Similarly, a significant body of experimental evidence indicates that peri-implant mucositis is also caused by the accumulation of bacterial biofilms (dental plaque) at the soft tissue–implant interface [28,29,30,31]. In addition, the ensuing local inflammatory response is being considered as a key in the pathogenesis of the peri-implant diseases. In general, microbial cells in mature biofilms are more resistant to antibiotics compared to those in a planktonic phase, since biofilms act as a protective barrier by limiting penetration of neutrophils, antibodies, or antimicrobial factors into a deeper area of biofilms [32,33,34]. Thus, optimal daily biofilm removal with adequate supportive periodontal therapy effectively prevents peri-implant mucositis, thereby decreasing the risk for peri-implantitis.

## 4. Current Knowledge of Oral Microbiology (Biofilms) Associated with Peri-Implant Mucositis

Previous studies showed that representative periodontopathic bacteria in periodontal pockets of residual teeth are highly associated with their presence in peri-implant pockets, due to the similarity of the peri-implant and periodontal sulcus environments [35,36,37,38,39]. Therefore, those representative periodontopathic bacteria, *Porphyromonas gingivalis*, *Treponema denticola*, *Tannerella forsythensis*, and *Aggregatibacter actinomycetemcomitans* [40,41,42], were considered as a risk indicator for peri-implant mucositis [43,44]. Yet, there are increasing reports showing the disparity of microbiota between peri-implant and periodontal diseases depending on the detection method of the microbiome and the range of species targeted [37,45]. For example, Koyanagi et al. reported that the biofilm in peri-implantitis showed a more complex microbial composition when compared with periodontitis [46]. They observed that *Fusobacterium* spp. and *Streptococcus* spp. were predominant in both peri-implantitis and periodontitis sites, while *Parvimonas micra* was exclusively detected in peri-implantitis [46]. By evaluating the whole microbiome via genomic sequencing, more uncultivable bacteria such as asaccharolytic anaerobic Gram-positive and Gram-negative rods as well as opportunistic microorganisms including enteric rods and *Staphylococcus aureus* were found in peri-implantitis sites, which were not frequently identified in teeth with periodontitis or healthy implants [45].

Additionally, previous microbiological studies assessed the composition of microbiota around implants and natural teeth. Interestingly, there were no statistical differences in the bacterial population nor microflora around implants and antagonist natural teeth [47]. However, some major periodontopathic microorganisms of red complex (e.g., *P. gingivalis*) and orange complex (e.g., *Prevotella intermedia*) were detected significantly more from implants affected by peri-implantitis than those not affected [47]. It indicates that the prognosis of peri-implant diseases can be achieved by detecting those periodontopathic microorganisms from implants.

## 5. Current Diagnosis and Treatment of Peri-Implant Diseases

Despite the fact that early diagnosis and accurate identification of risk factors are extremely important to effectively prevent peri-implant diseases, current systematic reviews revealed that a uniform classification and diagnostic methodology for peri-implantitis are lacking [48,49]. The 2017 World Workshop on the classification of periodontal and peri-implant diseases and conditions provided specific criteria to accurately define peri-implant status [50]. However, the diagnosis of peri-implant disease is heavily relying on clinical and radiographic data, which are neither sufficient nor sensitive to detect the disease at the initial phase [42]. For example, bleeding on probing, an indicator of inflammation in the peri-implant mucosa, has been considered a key clinical measure to distinguish between peri-implant health and disease. However, the case definition for peri-implantitis varied significantly between studies (e.g., probing depth and bleeding on probing), resulting in inconsistent distinction between health and disease [51]. Notably, there is no microbiological criterium in diagnosing peri-implant disease yet, although microbial infection at the interface of gingival tissue and implant is one of the primary causes of peri-implant diseases.

While the lack of regular supportive therapy in patients with peri-implant mucositis can escalate the risk for onset of peri-implantitis, there is no documented protocol available for the primary prevention of peri-implant mucositis. A variety of methods including air-powder abrasion, saline wash, citric acid application, laser therapy, peroxide treatment, ultrasonic/manual debridement, and application of topical medication have been applied to attempt to decontaminate the peri-implant sites. However, a definite gold standard could not be identified yet [52]. It has been shown that experimental peri-implant mucositis was significantly reversed via three weeks of professionally administered plaque removal and reinstitution of oral hygiene practices [29], indicating that the resolution of peri-implant mucositis is achievable. Therefore, the elimination of the plaque-biofilm from the implant surface should be the prime objective when treating peri-implantitis [52]. Indeed, patient-administered mechanical plaque control with manual or powered toothbrushes appeared to be an effective preventive measure. Additionally, professional interventions such as oral hygiene practices and mechanical debridement revealed a reduction in clinical signs of inflammation. In contrast, adjunctive measures (e.g., antiseptics, local and systemic antibiotics, air-abrasive devices) did not significantly improve the efficacy of professionally administered plaque removal procedures in reducing clinical signs of inflammation [53]. Since the failure of treating peri-implant mucositis may lead to progression of the peri-implant lesion or the bone loss that requires surgical therapy [52,54], a novel method for accurately diagnosing peri-implant mucositis at an early phase in conjunction with professionally administered plaque removal procedures is warranted.

## 6. Fluorescence-Based Early Detection of Peri-Implant Diseases

While early detection of peri-implant mucositis is critical to effectively prevent further progression to peri-implantitis, current peri-implant health diagnosis (e.g., bleeding on probing) fails to adequately predict disease progression [55]. As such, a need for point-of-care diagnosis has been described by many researchers and mirrors the needs of daily clinical practice. Fluorescence-based technology has the potential to be applied for the diagnosis and effective removal of plaques. Since light (photons) can propagate through the crystalline enamel and dentine tubules, changes in the mineral contents of teeth can be measured using light-scattering phenomena [56,57]. For instance, once the hard tissue is demineralized, the ultrastructure of enamel and dentine are changed; thereby, a distinct optical signal is garnered due to the modified light-tissue interactions [57,58,59]. In addition, some biological tissues can absorb and re-emit specific light wavelengths [60,61]. Thus, this technology can be used to assess the amount of bacteria or their metabolic activity.

Indeed, the applicability of fluorescence spectroscopy for the detection of calculus and plaque has been confirmed by several research groups using wavelengths from 360 up to 580 nm for excitation and different filters for the emission spectra [62,63,64,65]. For example, red and green autofluorescence glow of plaque has been observed from both in vitro and in vivo research studies [66,67,68,69]. Additionally, fluorescence glow around and on dental implants has been described and explained as a result of plaque, bacteria-contaminated calculus, and pentosidine [70]. It is worth noting that specific pathogenic bacteria could be diagnosed and differentiated from mature biofilms by detecting orange-to-red fluorescence porphyrins produced by peri-implant pathogens *P. gingivalis* and *A. actinomicetemcomitans*. As discussed earlier, microbiome composition is distinct between the implant with and without peri-implantitis. Implants affected by peri-implantitis harbor more red and orange complex bacteria. Therefore, utilizing fluorescence methodology may be a pragmatic way of diagnosing the potential incidence of peri-implantitis without a more complicated analysis method such as microbiome analysis or microCT.

**Figure 1:**
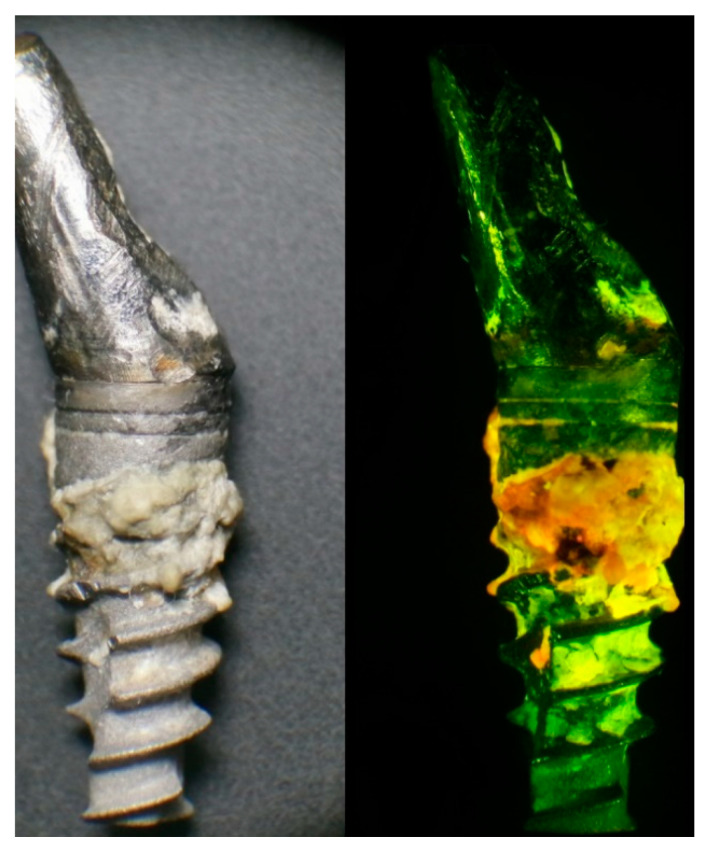
A failed implant after its removal due to peri-implantitis with significant plaque accumulation under natural light (**left**). Under UV headlight and special-filter loupes, plaque and active bacteria become visible in orange and red fluorescence (**right**). This may enable a proper differential diagnosis of peri-implant disease and facilitates accurate plaque removal.

Figure 1 shows an implant that failed due to peri-implantitis under natural and ultraviolet (UV) lights. As shown, plaque and active bacteria become distinct under UV light, which may enable proper differential diagnosis of peri-implant disease and facilitates accurate plaque removal. Furthermore, a fluorescence-based detection method can be used for not only the detection before the treatment but also the confirmation of successful debridement after the treatment. Completion of the biofilm elimination could be confirmed by visually inspecting the disappearance of the fluorescent from affected surfaces. Collectively, utilization of the fluorescence property of bacteria for point of care identification is a highly attractive new methodology with a wide spectrum of applications.

## 7. New Diagnostic Protocols

**Figure 2:**
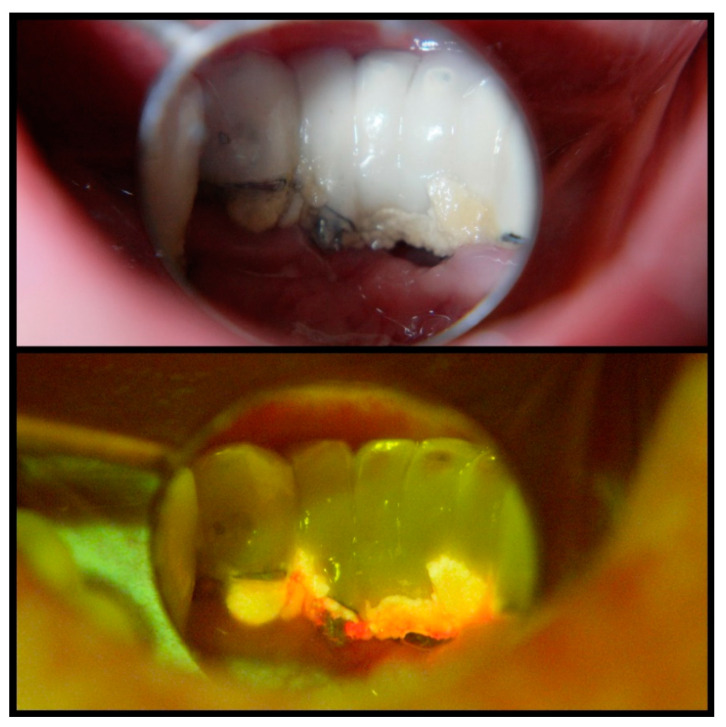
Intraoral clinical view of a mandibular anterior implant-supported restoration under regular operating light, indicating substantial plaque accumulation on the lingual aspect (**upper panel**). A UV headlight and special-filter loupes reveal active bacteria through orange and red fluorescence (**lower panel**) and allow for better detection and removal of plaque. Such a hands-free device does not limit the clinician during clinical treatment procedures.

Considering the potential use of a fluorescence-based approach, this with proper modification can be applied to improve the diagnosis of plaque biofilms and/or complete disinfection of biofilm contaminated dental implants. For example, rapid, precise but in-situ detection of plaque biofilms can be achieved by exclusively using fluorescence light excitation, which is not feasible with current traditional microbes detection methods such as plating and PCR assays. In particular, fluorescence-based technology through headlights combined with special-filtered dental loupes serve as a hands-free solution not just during the diagnostic phase and biofilm detection, but even more so during treatment for effective removal of biofilm. For example, an intraoral clinical view of a mandibular anterior implant-supported restoration under regular operating light shows substantial plaque accumulation on the lingual aspect (Figure 2, upper panel). It is more evident under a UV headlight with special-filter loupes, revealing active bacteria through orange and red fluorescence (Figure 2, lower panel). This allows for better detection and removal of plaque without limiting the clinician during clinical treatment procedures. Furthermore, this technology could be utilized to detect subgingival plaque/calculus build-ups that are not easily visible with naked eyes. The calibrated intensity of the fluorescence signal from such a portable fluorescence-based dental loupe and the number of bacteria or amount of molecules from pathogenic bacteria need to be determined in vitro and in vivo for these purposes. This fluorescence-based methodology is a simple technique that only requires basic training for visual examination. Notably, there is no known potential risk of the described technology up to date. Previous studies in examining cancerous lesions of the oral cavity [60] and bacterial metabolites associated with caries [71] using this fluorescence-based device displayed superior accuracy levels compared to clinical examination. Thus, such technology has the potential to fundamentally change clinical care with respect to plaque biofilm detection and removal, thereby enhancing clinical success and survival of endosseous dental implants in the long term.

## 8. Concluding Remarks

The current diagnosis modality on peri-implant diseases is relatively subjective and does not mirror the microbiological aspect of the diseases. Rapid detection and identification of pathogenic bacteria around implants are one of the most important factors to the prognosis of the incidence of peri-implant diseases that can result in severely painful and costly surgical procedures. Nevertheless, such microbial identification methods are not established yet in the clinical situation. Fluorescence-based clinical methods will help dental clinicians in rapidly and precisely detecting the microbiological risk-factor for peri-implant diseases and assessing clinical performance quantitatively.

## Data Availability

Not applicable.
